# Comparative Transcriptomic Analyses Reveal Potential Stp1 Regulatory Roles Independent of Sre1 in *Phaffia rhodozyma*

**DOI:** 10.3390/ijms262412008

**Published:** 2025-12-13

**Authors:** Marcelo Baeza, Melissa Gómez, Salvador Barahona, Maximiliano Coche-Miranda, Gabriela Apariz, Jennifer Alcaíno

**Affiliations:** 1 Departamento de Ciencias Ecológicas, Facultad de Ciencias, Universidad de Chile, Santiago 7800003, Chile; mbaeza@uchile.cl (M.B.); melissa.gomez@ug.uchile.cl (M.G.); maximiliano.coche@ug.uchile.cl (M.C.-M.); gabriela.apariz@ug.uchile.cl (G.A.); 2 Facultad de Ciencias, Universidad de Chile, Santiago 7800003, Chile; salvador@uchile.cl

**Keywords:** SREBP, Sre1, Stp1, RNA-Seq, yeast, sterols, gene regulation

## Abstract

Sterol regulatory element-binding proteins (SREBPs) regulate lipid homeostasis in mammals via sequential activation by the site-1 (S1P) and site-2 (S2P) proteases. In the yeast *Phaffia rhodozyma*, homologs of SREBP (Sre1) and S2P (Stp1) were identified, with Sre1 cleaved by Stp1 and involved in the regulation of sterol and carotenoid biosynthesis. Additional regulatory roles of S2P have been described in other organisms, but such functions remain unexplored in *P. rhodozyma*, a question addressed in this study. Transcriptomic analyses of Δ*sre1*, Δ*stp1*, and Δ*sre1*Δ*stp1* mutants were performed in both wild-type and Sre1-activated conditions. Potential genes regulated by Stp1 independently of Sre1 were identified, and their cellular roles were determined by KEGG mapping and Gene Ontology classification. As expected, most transcriptional changes in Δ*stp1* mutants reflected Sre1-mediated regulation. Notably, a subset of genes displayed differential expression independently of Sre1. These genes were linked to diverse aspects of cellular homeostasis, including metabolism, protein folding, ER stress response, and ribosome biogenesis. The transcriptomic analysis suggests that Stp1 regulates gene expression beyond the Sre1 transcription factor in *P. rhodozyma*, providing a framework for future studies to confirm and further explore these functions.

## 1. Introduction

Lipids are fundamental components of cellular membranes, functioning not only as structural elements but also as precursors of signaling molecules and as key modulators of energy balance and stress responses [1]. Their homeostasis requires a fine-tuned coordination of biosynthetic and degradative pathways, where the sterol regulatory element-binding protein (SREBP) pathway represents a central regulatory system broadly conserved across eukaryotes [2,3]. SREBPs are transcription factors synthesized as inactive precursors anchored to the endoplasmic reticulum (ER) through two transmembrane helices, exposing both the N-terminal transcription factor domain and the C-terminal regulatory domain to the cytoplasm [4]. In mammals, SREBPs interact with the ER-resident protein SCAP (SREBP cleavage-activating protein), which senses sterol levels and when sterol concentrations decrease, it escorts SREBPs to the Golgi apparatus. In the Golgi, SREBPs are sequentially processed by site-1 protease (S1P) and site-2 protease (S2P), releasing the N-terminal transcription factor domain that translocates to the nucleus to regulate sterol-responsive genes [5]. S2P proteases are widely conserved intramembrane metallopeptidases present in Bacteria, Archaea, and Eukarya [6,7]. In bacteria, S2P-mediated cleavage of anti-sigma factors releases σ-factors that activate adaptive responses to extracytoplasmic signals [8]. In eukaryotes, S2P not only activate SREBPs but also other membrane-bound transcription factors [7]. A well-characterized example is the Activating transcription factor 6 (ATF6), which triggers the unfolded protein response under ER stress [9]. Similarly, members of the CREB3 transcription factor family are activated via S1P/S2P mediated proteolysis, regulating diverse cellular processes such as ER and Golgi stress responses, metabolism, and secretion [7,10,11].

Two components of the SREBP pathway have been identified and functionally characterized in *Phaffia rhodozyma*: Sre1, the SREBP homolog [12], and Stp1, the S2P homolog [13]. *P. rhodozyma* is a basidiomycete yeast notable for its ability to synthesize carotenoids, particularly astaxanthin, a pigment of great commercial interest highly valuable in aquaculture, cosmetics, and nutraceuticals due to its pigmentation and antioxidant properties [14,15,16]. This species was originally isolated from tree exudates in cold mountainous regions of the Northern Hemisphere, including Japan and Alaska, in the 1960s, and later recovered from diverse geographic locations worldwide [17]. Its ability to produce astaxanthin has been proposed to confer advantages, enabling its survival under oxidative or other environmental stress, as carotenoids provide protection against reactive oxygen species [18]. These ecological and metabolic features make *P. rhodozyma* a valuable system for studying carotenoid biosynthesis, stress physiology, and lipid-associated metabolic pathways. Notably, carotenogenesis in this yeast is regulated by the SREBP pathway, through Sre1 regulation of the mevalonate pathway, including the regulation of cytochrome P450 enzymes, being the crosstalk between the biosynthesis of carotenoids and sterols [19]. Interestingly, *P. rhodozyma* lacks a SCAP homolog (the sterol sensor protein), suggesting that the processing of Sre1 in this yeast depends on a still unknown SCAP-independent mechanism [20].

The SREBP pathway has been described in a few fungi and has been shown to be more diverse. Sterol regulation in *Saccharomyces cerevisiae* relies on the Upc2/Ecm22 transcription factors rather than SREBPs [21], while in *Schizosaccharomyces pombe*, the SREBP ortholog Sre1 is activated by the Golgi Dsc E3 ligase complex rather than by S1P/S2P cleavage [22,23]. In the basidiomycete *Cryptococcus neoformans*, the Sre1 transcription factor is processed by Stp1, a functional homolog of S2P [24]. Interestingly, deletion of *STP1* in *C. neoformans* altered the expression of hundreds of genes, some independently of Sre1, suggesting that fungal S2Ps may also extend their regulatory role beyond a single transcription factor [24]. Based on these observations, we hypothesize that Stp1 in *P. rhodozyma* may also influence additional transcriptional programs beyond its role in activating Sre1. To test this hypothesis, we performed comparative transcriptomic analyses of *STP1*-deficient strains derived from distinct *P. rhodozyma* genetic backgrounds.

## 2. Results

### 2.1. Comparative Transcriptomic Analysis of Δsre1 and Δstp1 Mutants in P. rhodozyma

**Figure 1:**
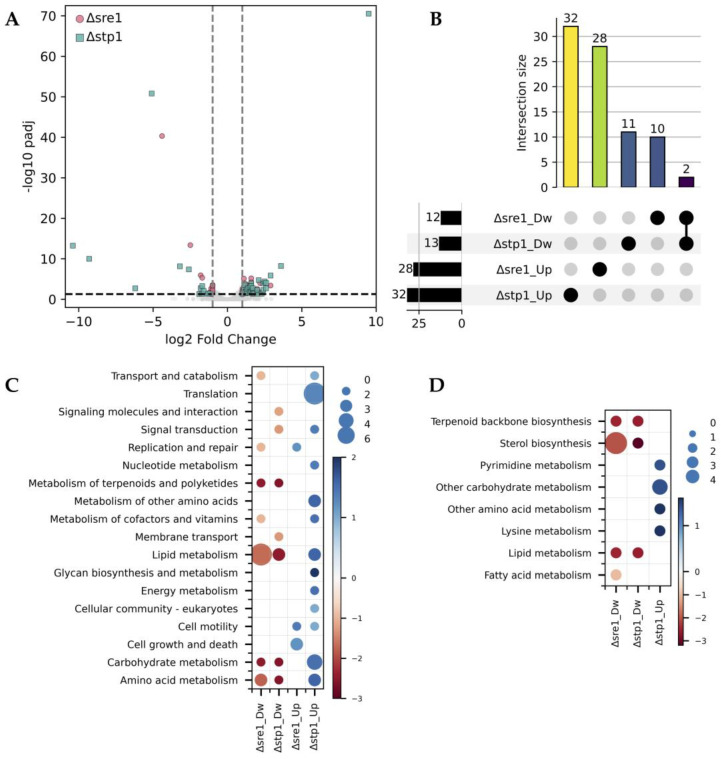
Differential gene expression in Δ*sre1* and Δ*stp1* mutants relative to a wild-type genetic context. (**A**) Volcano plot showing the distribution of DEGs for Δ*sre1* (pink circles) and Δ*stp1* (green squares) relative to the wild-type genetic background (strain WTF). Vertical dashed lines indicate the ± 1 log_2_ fold-change threshold, and the horizontal dashed line marks the significance cutoff (padj ≤ 0.05). (**B**) UpSet plot illustrating the unique and shared DEGs between Δ*sre1* and Δ*stp1* mutants relative to strain WTF. Numbers at the left or above the bars indicate the number of DEGs in each category. (**C**) KEGG pathway (P2) distribution of DEGs in each mutant. (**D**) KEGG module (M2) distribution of DEGs in each mutant. In (**C**,**D**), circle size represents the number of DEGs in each category, and the color scale indicates the average log_2_ fold-change. Dw: downregulated genes; Up: upregulated genes.

To investigate whether Stp1 has regulatory effects independent of Sre1 in *P. rhodozyma*, we compared the transcriptomic profiles of Δ*sre1* and Δ*stp1* mutants relative to the CBS.*FLAG*.*SRE1* strain (hereafter referred to as WTF). While both mutants displayed relatively few DEGs (<50 each), the vast majority were unique to each strain, with only two downregulated genes shared between both transcriptomes (Figure 1A,B). The distribution of DEGs also differed according to their classification across KEGG metabolic pathways and functional modules (Figure 1C,D). In both mutants, alterations were observed in cellular processes beyond the canonical role of the SREBP pathway in lipid homeostasis. Notably, despite the relatively low number of DEGs, several pathways were represented exclusively in Δ*stp1*, including *Membrane transport*, *Signal transduction*, and *Signaling molecules* and *interactions* among downregulated genes, and *Translation* and *Carbohydrate metabolism* among upregulated ones. These observations suggest that Stp1 is involved in transcription regulation beyond canonical Sre1 regulation, influencing broader aspects of cellular regulation, such as nutrient signaling and protein synthesis, independently of Sre1. Although a similar reasoning can also be applied to DEGs found exclusively in Δ*sre1*, this is not expected because available evidence indicates that Sre1 is not activated in the absence of Stp1 (at least not detectable by Western blot) [13]. It is possible that the complete loss of Sre1 in the Δ*sre1* mutant may have broader physiological consequences than only its inability to be activated in the Δ*stp1* mutant (see Section 3).

### 2.2. Analysis of Transcriptional Response of the P. rhodozyma Δsre1Δstp1 Mutant Suggests Sre1-Independent Roles of Stp1

**Figure 2:**
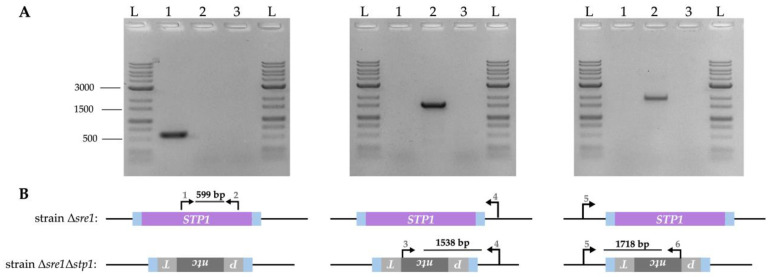
*STP1* gene replacement in *P. rhodozyma* genome. (**A**) PCR analysis using genomic DNA from strains Δ*sre1* (lane 1), Δ*sre1*Δ*stp1* (lane 2), and a negative control without DNA (lane 3). The GeneRuler 1 kb Plus DNA Ladder (L) was used as a molecular weight marker. (**B**) Schematic representation of the amplified fragments provided beneath each gel, indicating the expected sizes for each strain. Primers are shown as arrows and numbered according to Table S1. *STP1* gene (purple), nourseothricin resistance cassette (gray), and flanking regions of the *STP1* locus to promote homologous recombination (blue).

A valuable comparison to determine which transcriptional effects of Stp1 are independent of Sre1 is to compare Δ*sre1* versus Δ*sre1*Δ*stp1* transcriptomic results, to isolate the effects of *STP1* deletion from any influence that the non-activated Sre1 precursor might still exert. For that, the double mutant strain (strain Δ*sre1*Δ*stp1*) was generated by transforming the Δ*sre1* strain with a recombination module excised from plasmid pBS-Δg*STP1^nat^*. This module consisted of 715 bp upstream and 620 bp downstream flanking regions of the *STP1* locus to promote homologous recombination, and a nourseothricin resistance cassette between them. The correct replacement of *STP1* with the resistance cassette was verified by PCR analysis using primer sets designed to confirm the absence of the *STP1* gene and both junctions of the integration event (Figure 2).

**Figure 3:**
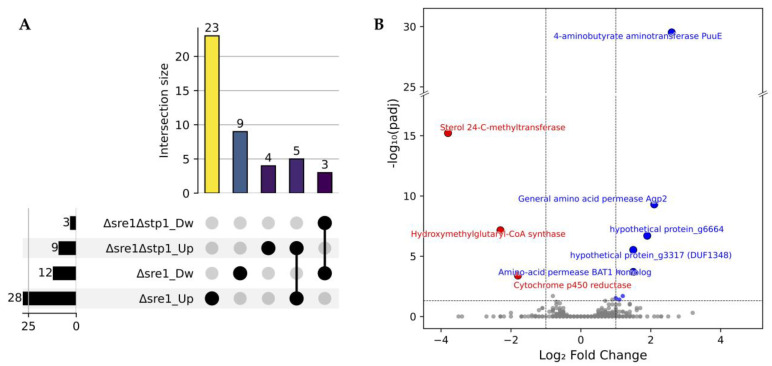
Differential gene expression in the double mutant strain Δ*sre1*Δ*stp1*. (**A**) UpSet plot illustrating unique and common DEGs in Δ*sre1*Δ*stp1* and Δ*sre1* mutants compared to strain WTF. Numbers at the left or above the bars indicate the number of DEGs in each category or intersection. (**B**) Volcano plot representing DEGs in Δ*sre1*Δ*stp1* relative to the wild-type genetic background (strain WTF). Vertical dashed lines indicate ± 1 log_2_ fold change threshold, and the horizontal dashed line marks the significance cutoff (padj ≤ 0.05). Dw: downregulated and Up: upregulated genes.

Differential expression analysis of the Δ*sre1*Δ*stp1* mutant relative to WTF revealed a small set of DEGs, comprising three downregulated and nine upregulated genes. Despite the limited number of DEGs, comparison of Δ*sre1*Δ*stp1* and Δ*sre1* relative to WTF revealed both subsets of DEGs that were unique to each mutant as well as shared ones (Figure 3A). The DEGs exclusive to each mutant suggest regulatory functions independent of one another, which, in the case of Δ*sre1*Δ*stp1*, included four unique upregulated genes.

Figure 3B shows the distribution of DEGs in Δ*sre1*Δ*stp1* relative to WTF, highlighting the top up- and downregulated genes. The downregulation of *sterol 24-C-methyltransferase*, *hydroxymethylglutaryl-CoA synthase*, and *cytochrome P450 reductase* in the Δ*sre1*Δ*stp1* mutant is consistent with their identification as direct Sre1 targets in *P. rhodozyma* [19], confirming that the loss of both regulators compromises canonical SREBP-mediated sterol biosynthesis. In contrast, the upregulated genes were mainly associated with amino acid metabolism and transport, such as *4-aminobutyrate aminotransferase (PuuE)*, the *general amino acid permease Agp2*, and a *BAT1 amino acid permease homolog*, suggesting additional regulation by Stp1 beyond sterol homeostasis.

### 2.3. Transcriptomic Signatures Comparison in Two Sre1-Activated Contexts in P. rhodozyma, cyp61^−^ Versus Sre1N Strains

In *P. rhodozyma*, Sre1 is activated at basal levels in the wild-type under the tested culture conditions and becomes significantly activated when sterol synthesis is perturbed [12,13]. Consequently, not all potential targets of Sre1, and probably of Stp1, can be detected in WTF under these conditions. To uncover a broader set of regulated genes, transcriptomic analyses under conditions where Sre1 is activated would be more informative. One such context is the previously described *cyp61*^−^ mutant [25], in which disruption of ergosterol biosynthesis leads to Sre1 activation, resulting in a distinctive Sre1-dependent transcriptional signature [12,19].

**Figure 4:**
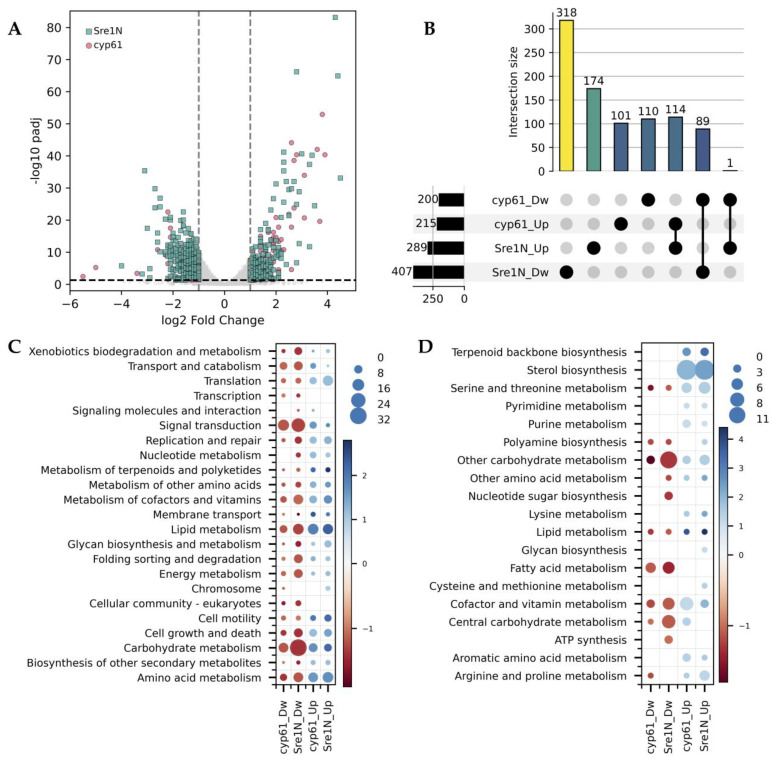
Differential gene expression in strains *cyp61*^−^ and Sre1N relative to a wild-type genetic context. (**A**) Volcano plot showing DEGs in *cyp61*^−^ (pink circles) and Sre1N (green squares) relative to the wild-type genetic background (strain WTF). Vertical dashed lines indicate ±1 log_2_ fold change threshold, and the horizontal dashed line marks the significance cutoff (padj ≤ 0.05). (**B**) UpSet plot showing unique and common DEGs in *cyp61*^−^ and Sre1N relative to strain WTF. Numbers at the left or above the bars indicate the number of DEGs in each category or intersection. (**C**) KEGG pathway (P2) distribution of DEGs in each mutant. (**D**) KEGG module (M2) distribution of DEGs in each mutant. In (**C**,**D**), circle size represents the number of DEGs in each category, and the color scale indicates the average log_2_ fold-change. Dw: downregulated and Up: upregulated genes.

To further validate this approach, the transcriptional profile of strain *cyp61*^−^ was compared with that of the Sre1N strain, which constitutively expresses the active form of Sre1 [12]. Both mutants exhibited extensive transcriptional differences relative to WTF (415 DEGs in *cyp61*^−^ and 696 DEGs in Sre1N), including both up- and downregulated DEGs (Figure 4A). Several DEGs were exclusive to each mutant, with a higher proportion of exclusive DEGs in Sre1N (78% and 60% exclusive down- and upregulated genes, respectively) compared to *cyp61*^−^ (55% and 47% exclusive down- and upregulated genes, respectively) (Figure 4B). Among the shared DEGs, 89 were downregulated and 114 were upregulated in both mutants, suggesting that nearly half of the transcriptional changes observed in the *cyp61*^−^ mutant can be attributed to Sre1 activity.

As shown in Figure 4C,D, the distribution of DEGs across KEGG pathways and modules was similar in both mutants, with Sre1N generally showing a higher number of DEGs and greater average log_2_ fold change than *cyp61*^−^. Differences were observed in *Nucleotide metabolism* and *Chromosome* pathways, where downregulated and upregulated DEGs, respectively, were only detected in Sre1N. In contrast, both mutants were similar in *Lipid metabolism* and *Sterol biosynthesis*, particularly among upregulated DEGs, consistent with Sre1 activity [19].

### 2.4. Comparative Transcriptomic Analysis of Δsre1 and Δstp1 Mutations in a cyp61^−^ Genetic Background of P. rhodozyma

**Figure 5:**
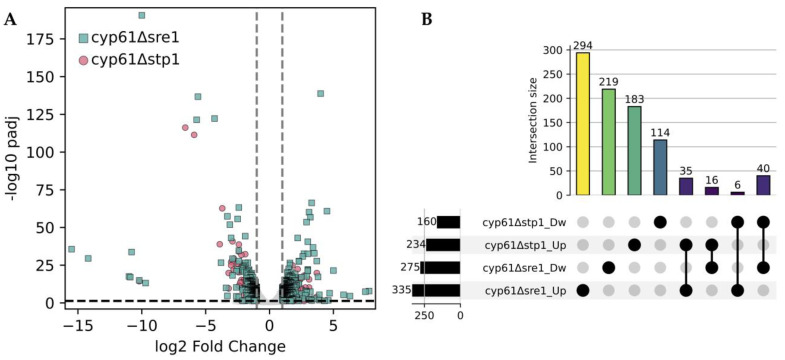
Differential gene expression in *cyp61*^−^*Δsre1* and *cyp61*^−^*Δstp1* mutants relative to the *cyp61*^−^ genetic context. (**A**) Volcano plot showing the distribution of DEGs for *cyp61*^−^Δ*sre1* (green squares) and *cyp61*^−^Δ*stp1* (pink circles) relative to strain *cyp61*^−^. Vertical dashed lines indicate ±1 log_2_ fold change threshold, and the horizontal dashed line marks the significance cutoff (padj ≤ 0.05). (**B**) UpSet plot illustrating unique and common DEGs in *cyp61*^−^Δ*sre1* and *cyp61*^−^Δ*stp1* mutants relative to strain *cyp61*^−^. Numbers at the left or above the bars indicate the number of DEGs in each category or intersection. Dw: downregulated and Up: upregulated genes.

The transcriptomes of strains *cyp61*^−^Δ*sre1* and *cyp61*^−^Δ*stp1* were analyzed and compared with that of their parental strain *cyp61*^−^, revealing distinct expression patterns for each mutant (Figure 5A). Both mutants exhibited a higher number of DEGs in the *cyp61*^−^ than in the wild-type background, including DEGs unique to each mutant as well as those shared between them (Figure 5B). These results support the existence of genes co-regulated by Stp1 and Sre1, while also suggesting a set of genes likely regulated exclusively by Stp1, which may be among the 114 downregulated and 183 upregulated DEGs exclusive to the *cyp61*^−^Δ*stp1* mutant.

### 2.5. Stp1-Dependent Regulation Beyond Sre1 Activation in P. rhodozyma

As with the comparisons performed in the wild-type genetic background, a double mutant (*cyp61*^−^Δ*sre1*Δ*stp1*) was constructed to compare its transcriptional changes with those of *cyp61*^−^Δ*sre1*, thereby isolating the effects of the *STP1* deletion from any potential Sre1-dependent activity. To construct this strain, the *cyp61*^−^Δ*sre1* strain was transformed with the recombination module excised from plasmid pBS-Δg*STP1^nat^*, in which the *STP1* gene was replaced by the nourseothricin resistance cassette. Successful gene replacement was confirmed by PCR analysis (Figure S1).

**Figure 6:**
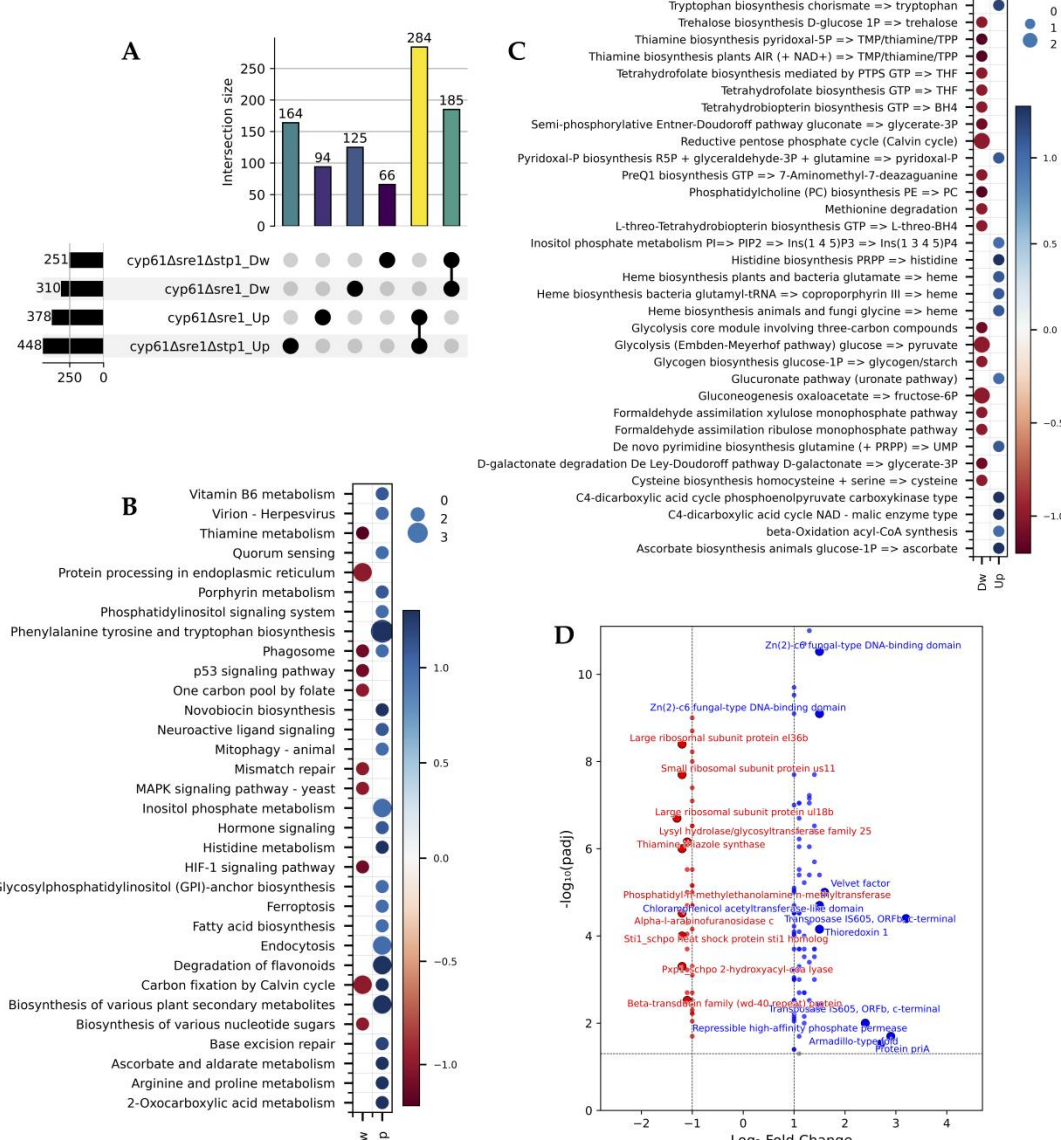
Effects of the *STP1* gene deletion in a *cyp61*^−^ genetic context lacking *SRE1.* (**A**) UpSet plot illustrating unique and common DEGs in *cyp61*^−^Δ*sre1* and *cyp61*^−^Δ*sre1*Δ*stp1* mutants relative to strain *cyp61*^−^. Numbers at the left or above the bars indicate the number of DEGs in each category or intersection. (**B**) KEGG pathway (P3) distribution of DEGs in *cyp61*^−^Δ*sre1*Δ*stp1*. (**C**) KEGG module (M3) distribution of DEGs in *cyp61*^−^Δ*sre1*Δ*stp1*. In B and C, circle size represents the number of DEGs in each category, and the color scale indicates the average log_2_ fold-change. (**D**) Volcano plot showing the distribution of DEGs observed exclusively in *cyp61*^−^Δ*sre1*Δ*stp1* relative to strain *cyp61*^−^. Vertical dashed lines indicate ±1 log_2_ fold change threshold, and the horizontal dashed line marks the significance cutoff (padj ≤ 0.05). The top 10 downregulated (red) and upregulated (blue) genes are labeled. Dw: downregulated genes; Up: upregulated genes.

In the comparison of DEGs in the *cyp61*^−^Δs*re1*Δ*stp1* and *cyp61*^−^Δ*sre1* mutants relative to their parental strain *cyp61*^−^, the number of shared up- and downregulated DEGs exceeded those unique to each mutant (Figure 6A), in contrast to the patterns observed under the wild-type background. This result indicates that under the activated genetic context provided by the *cyp61*^−^ mutation, most transcriptional changes are likely attributable to canonical Stp1-mediated regulation via Sre1 activation. Notably, 66 downregulated and 164 upregulated genes were detected exclusively in *cyp61*^−^Δ*sre1*Δ*stp1*, suggesting that their expression is altered when Stp1 is absent but restored when Stp1 is present in *cyp61*^−^Δ*sre1*. These 230 genes therefore represent potential targets regulated by Stp1 independently of Sre1.

Classification of these candidate genes into KEGG metabolic pathways (Figure 6B) and modules (Figure 6C) yielded 32 and 33 categories, respectively. Using a threshold of ≥1.3 for the average |log_2_FC| revealed nine pathways, including *Histidine metabolism*, *Novobiocin biosynthesis*, *Ascorbate and aldarate metabolism*, and *2-oxocarboxylic acid metabolism*. Similarly, four modules met the threshold: *Histidine biosynthesis (PRPP → histidine)*, *Ascorbate biosynthesis in animals (glucose-1P → ascorbate)*, the *C4-dicarboxylic acid cycle (NAD–malic enzyme type)*, and the *C4-dicarboxylic acid cycle (phosphoenolpyruvate carboxykinase type)*.

Among the candidate genes, 135 encoded proteins with BLASTp (version 2.17.0) hits and were classified according to their |log_2_FC| values as strong (≥1.5; 11 genes), moderate (1.4–1.3; 20 genes), or low (1.2–1.0; 103 genes). All strongly regulated genes, and 19 of the 20 moderately regulated ones, were upregulated in the *cyp61*^−^Δ*sre1*Δ*stp1* mutant. Figure 6D shows the volcano plot of these 135 genes, highlighting the top ten up- and downregulated ones.

Several of the upregulated genes suggest potential roles of Stp1 in regulatory processes beyond regulation of sterol homeostasis. These included two *Zn(2)-C6 fungal-type DNA-binding domain proteins* a *Velvet factor*, a *repressible high-affinity phosphate permease* and *Thioredoxin 1*. Other upregulated genes included those encoding proteins with *Armadillo-type folds* and *PriA-like domains*. Interestingly, two *transposase IS605 ORFb, C-terminal* genes were also upregulated. Among the top downregulated genes identified exclusively in the *cyp61*^−^Δ*sre1*Δ*stp1* mutant, several ribosomal proteins (*ul18b*, *us11*, and *el36b*), a *Sti1 homolog*, *thiamine thiazole synthase*, and *phosphatidyl-N-methylethanolamine N-methyltransferase* were repressed.

A total of 95 genes among the 230 candidates could not be identified by BLASTp (yielding hits with hypothetical proteins or no BLAST hits) and were therefore classified as “hypothetical proteins”. Nevertheless, conserved domains were predicted for several of them (Table S2), suggesting potential functional roles yet to be characterized. The most frequently predicted domains included *WSC* (*putative carbohydrate-binding*) *domain*, *major facilitator superfamily*, *tetratricopeptide repeat*, *centromere kinetochore component*, *core histones (H2A/H2B/H3/H4)*, *HAMP domain*, and *mitochondrial carrier protein*. When considering only strongly regulated hypothetical proteins, the most common domains were *centromere kinetochore component*, *major facilitator superfamily*, *fungal Zn(2)-Cys(6) binuclear cluster domain*, and *hydantoinase/oxoprolinase*.

To gain a complementary perspective on the functions of the 230 DEGs exclusive to *cyp61^−^Δsre1Δstp1*, but not altered in *cyp61^−^Δsre1*, GO enrichment analyses were performed. This analysis revealed consistent patterns across the three ontologies: Biological Process (BP), Molecular Function (MF), and Cellular Component (CC), using both clusterProfiler and TopGO (Table 1). In BP, the enriched GO terms were primarily associated with downregulated genes, including *glycolytic process* and *ATP synthesis coupled proton transport*. In MF, the most enriched GO terms, also driven by downregulated genes, were *Structural constituent of ribosome*, *Proton-transporting ATP synthase activity*, and *Unfolded protein binding*. Finally, in CC, enriched GO terms included *Ribosome* (downregulated genes) and *Nucleolus* (upregulated genes). Overall, these observations suggest that the downregulated genes primarily impact essential cellular processes, particularly protein synthesis.

**Figure 7:**
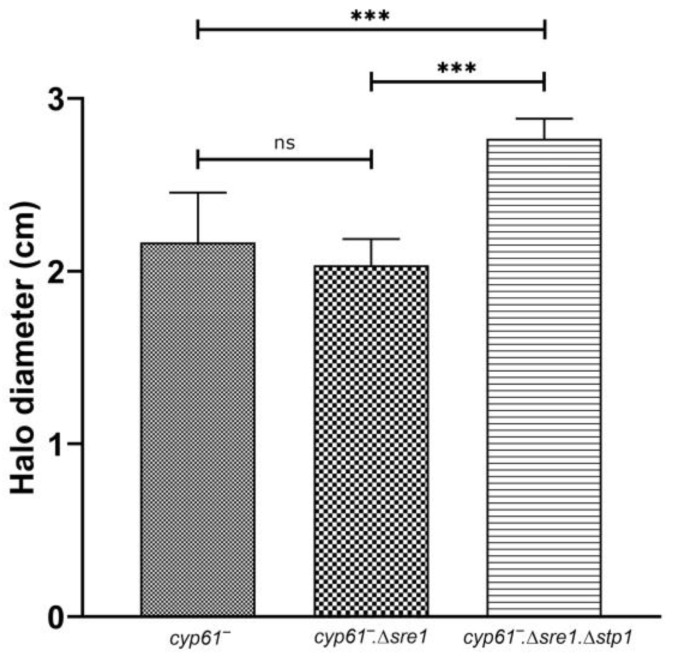
DTT sensitivity assay. Cell lawns of *cyp61*^−^, *cyp61*^−^Δ*sre1* and *cyp61*^−^Δ*sre1*Δ*stp1* strains were prepared on YM-1.5% agar plates. A sterile filter disk was placed at the center of each plate, and 10 µL of 1.0 M DTT were applied directly onto the disk. Plates were incubated at 22 °C for two days, and halo diameters were recorded. Data represent the mean of three independent replicates, and error bars indicate standard deviation. Statistical analysis was performed using two-way ANOVA followed by Tukey’s post hoc test. *** *p* < 0.001, (ns: not significant).

Finally, considering that several DEGs among the differentially expressed genes in the *cyp61*^−^Δ*sre1*Δ*stp1* mutant that were independent of Sre1 were associated with protein processing at the ER, protein synthesis, and stress adaptation, among others, we tested if Stp1 could contribute to cellular response to ER stress. For this, we performed a dithiothreitol (DTT) sensitivity assay, as DTT affects protein folding in the ER by disrupting disulfide bond formation [26]. Notably, the *cyp61*^−^Δ*sre1*Δ*stp1* strain displayed increased sensitivity to DTT compared to its parental *cyp61*^−^ strain and to *cyp61*^−^Δ*sre1* (Figure 7 and Figure S3), supporting that Stp1 participates in the response to ER stress independent from Sre1.

## 3. Discussion

In this work, we performed a comparative transcriptomic analysis to evaluate whether Stp1 contributes to gene regulation beyond its established role through Sre1 activation in *P. rhodozyma*. Using wild-type and sterol-altered genetic backgrounds, we identified candidate genes and pathways that may reflect Sre1-independent functions of Stp1 in this yeast. To our knowledge, *P. rhodozyma* represents the only basidiomycete, other than *C. neoformans*, in which a functional homolog of Stp1 (S2P analog) has been characterized [20], making it a useful model to explore potential Sre1-independent regulatory functions of this protease within this division.

### 3.1. Stp1 Influences Transcriptional Programs Beyond Sterol Regulation Under a Wild-Type Background

The total number of DEGs in both mutants (Δ*sre1* and Δ*stp1*) was relatively small under a wild-type genetic context, with only two downregulated genes (*Hydroxymethylglutaryl-CoA synthase* and *Sterol 24-C-methyltransferase*) shared between them, which are direct Sre1 targets, as previously determined by ChIP-exo experiments [19]. The remaining DEGs were uncommon and affected distinct biological processes, which may reflect the cellular consequences of losing Sre1 activity, where disruption of lipid and sterol homeostasis could trigger compensatory transcriptional responses extending to both Sre1-dependent and independent genes. These observations are consistent with previous findings in *C. neoformans*, where transcriptomic analysis under alkaline growth conditions showed that deletion of *SRE1* altered nearly one-quarter of the transcriptome, demonstrating the broad impact of Sre1 loss [27].

In the Δ*stp1* mutant, the identification of pathways unrelated to sterol metabolism suggests that Stp1 may also regulate other cellular functions beyond its canonical role in Sre1 activation, possibly through the activation of additional, yet unknown, transcription factors or signaling components. This idea is reinforced by studies in *C. neoformans*, where deletion of *STP1* and *SRE1* also induced transcriptional changes beyond sterol and lipid metabolism, suggesting that Stp1 may regulate other transcription factors or pathways independently of Sre1 in this yeast [24]. Transcriptomic results from the Δ*sre1*Δ*stp1* mutant supports the notion that Stp1 may influence pathways beyond sterol homeostasis. Among upregulated genes in this double mutant is a potential general amino acid permease Agp2, which in *Saccharomyces cerevisiae* plays a central role in polyamine transport [28] influencing the expression of transporters and drug resistance genes [29]. Therefore, the induction of a potential Agp2-like permease in *P. rhodozyma* suggests that Stp1 may influence amino acid uptake and sensing. Likewise, the upregulation of a BAT1-like permease is notable, since BAT1 functions as a bidirectional amino acid transporter [30]. The higher expression of *4-aminobutyrate aminotransferases*, which transfers the amino group from *4-aminobutyrate* (GABA) to alpha-ketoglutarate to yield succinic semialdehyde and glutamate [31], suggests a link between nitrogen and carbon metabolism via GABA utilization for the TCA cycle. Taken together, the upregulation of these genes in the absence of both Stp1 and Sre1 may reflect compensatory responses connected to nitrogen and amino acid metabolism triggered by disrupted lipid homeostasis. Alternatively, it raises the possibility that Stp1 could indirectly influence amino acid regulatory networks, potentially through the proteolytic activation of additional membrane-bound transcription factors beyond Sre1.

### 3.2. Establishing cyp61^−^ as an Appropriate Sre1-Activated Model for Regulatory Comparisons

Given the limited number of DEGs detected under a wild-type background, we next analyzed transcriptional responses in a genetic context where Sre1 is activated, reasoning that Stp1-dependent effects might become more apparent under these conditions. Before assessing the effects of *STP1* deletion in this background, we first compared the transcriptional profiles of *cyp61*^−^ and Sre1N. Although this comparison had been reported previously [19], it was not performed using strains carrying the FLAG-tagged *SRE1* allele as in this work, making it necessary to re-evaluate these datasets to ensure an appropriate and internally consistent baseline. The transcriptional profile of *cyp61*^−^ strain used in this work was consistent with previous reports [19], indicating that the FLAG tag does not substantially alter Sre1 regulation and thus provides a valid reference for our analyses.

As expected, strain *cyp61*^−^ elicited a weaker transcriptomic response than Sre1N, which showed more and stronger transcriptional changes consistent with its constitutive and likely deregulated activity. These results suggest that the transcriptional changes observed in the *cyp61*^−^ mutant encompass those driven by Sre1N but in a more physiological context, supporting its use as a suitable background to identify genes regulated by Stp1 independently of Sre1.

### 3.3. Expanded Regulatory Potential of Stp1 Revealed in an Altered Sterol Level Context

The transcriptomic analyses performed in the *cyp61*^−^ background provided a clearer view of the potential Sre1-independent functions of Stp1, as Sre1 is activated under these conditions and differences attributable to Stp1 become more apparent. The transcriptomes of strains *cyp61*^−^Δ*sre1* and *cyp61*^−^Δ*stp1* revealed distinct expression patterns relative to the parental *cyp61*^−^ background, and both mutants showed a noticeably higher number of DEGs than in comparisons performed under the wild-type context. This increase likely reflects the activated state of the Sre1 pathway in *cyp61*^−^, which may amplify transcriptional differences caused by the absence of either Sre1 or Stp1. The substantial number of DEGs exclusive to *cyp61*^−^Δ*stp1* suggests that Stp1 influences additional pathways independently of Sre1. Although Sre1 is not activated in the Δ*stp1* mutant in the *cyp61*^−^ background [13], suggesting that no alternative protease can compensate for the absence of Stp1 to activate Sre1 under the tested conditions, there are still DEGs unique to the *cyp61*^−^Δ*sre1* strain. This observation reflects biological differences between Δ*sre1* and Δ*stp1* strains, as deleting *SRE1* removes the transcription factor entirely, whereas deleting *STP1* leaves the full-length, membrane-bound Sre1 present but unable to be processed by Stp1. The presence of the inactive Sre1 protein may still allow interactions with membrane components, chaperones, or other regulatory factors. Thus, even though both mutations impair Sre1 function as a transcription factor, the presence of the unprocessed Sre1 protein in Δ*stp1* may attenuate indirect transcriptional changes, whereas the complete absence of Sre1 in Δ*sre1* is more likely to elicit compensatory responses causing different transcriptional effects. For this reason, we used the double mutant to distinguish transcriptional effects that reflect the absence of Stp1 itself from those arising from the complete loss of Sre1. To explore this possibility more directly, we examined the double mutant *cyp61*^−^Δ*sre1*Δ*stp1*, reasoning that genes whose expression changes only when both regulators are absent, but not in *cyp61*^−^Δ*sre1*, would represent stronger candidates for Sre1-independent targets of Stp1. Notably, 230 genes (66 downregulated and 164 upregulated) were detected exclusively in the double mutant, indicating that their expression is altered only when Stp1 is absent but remains unchanged in *cyp61*^−^Δ*sre1*. These genes therefore represent the strongest candidates for Sre1-independent regulation by Stp1.

Among these 230 candidates, several functional signatures emerged that provide insight into the processes that may rely on Stp1 independently of Sre1. Analysis of KEGG pathways and modules suggest that Stp1 may act as an indirect regulator of amino acid metabolism, in redox balance, and central carbon metabolism. In particular, the prominence of histidine metabolism, an amino acid known to be critical for metal homeostasis and virulence in *Aspergillus fumigatus* [32], raises the possibility that Stp1 may contribute to adaptive responses. Likewise, the representation of ascorbate-related metabolism (vitamin C) suggests the involvement of alternative antioxidant systems, such as erythroascorbate, a structural and functional analog of vitamin C that supports oxidative stress defense in yeasts [33]. Increased representation of *2-oxocarboxylic acid metabolism* indicates a potential role of Stp1 in pathways involving α-ketoacids, which link central carbon metabolism and amino acid biosynthesis [34]. Finally, the occurrence of enzymes from the C4-dicarboxylic acid cycle module points to a possible contribution of Stp1 to energy and cofactor homeostasis, since malic enzyme and phosphoenolpyruvate carboxykinase influence NAD(P)H and ATP balance, respectively [35,36].

Several of the upregulated genes suggest potential roles of Stp1 in regulatory processes beyond regulation of sterol homeostasis. These included two *Zn(2)-C6 fungal-type DNA-binding domain proteins* and a *Velvet factor*, which are linked to transcriptional regulators and consistent with the idea that Stp1 may influence additional transcriptional programs as proposed for *C. neoformans* [24]. The induction of a *repressible high-affinity phosphate permease* suggests that Stp1 may influence phosphate uptake, a macronutrient essential for fungal growth and biosynthetic processes such as nucleic acid and phospholipid production [37]. The upregulation of *Thioredoxin 1* further points to a possible role in redox balance, as thioredoxin systems are key players in redox homeostasis and oxidative stress defense [38]. Other upregulated genes included those encoding proteins with *Armadillo-type folds*, implicated in protein–protein interactions across diverse processes such as cytoskeleton and mitochondrial function regulation [39], and *PriA-like domains*, linked to DNA replication and recombination [40]. Interestingly, two *transposase IS605 ORFb*, *C-terminal* genes were also upregulated, pointing to possible contributions of Stp1-dependent regulation to genome plasticity, considering the role of transposable elements in fungal adaptation [41,42]. Among the top downregulated genes identified exclusively in the *cyp61*^−^Δ*sre1*Δ*stp1* mutant, several ribosomal proteins (*ul18b*, *us11*, and *el36b*) were repressed, suggesting that Stp1 may impact translation capacity. Since ribosome biogenesis and protein synthesis are tightly coupled to metabolism and growth [43,44], this repression points to a role of Stp1 in coordinating protein synthesis with metabolic state and nutrient availability. The repression of a *Sti1 homolog*, a co-chaperone involved in Hsp90 function [45], further suggests that Stp1 may influence protein folding, in line with the role of other S2P substrates in ER stress regulation [9,10]. Reduced expression of *thiamine thiazole synthase* suggests a possible role in vitamin B1 biosynthesis, a cofactor central to carbohydrate metabolism [46], while repression of *phosphatidyl-N-methylethanolamine N-methyltransferase* points to potential effects on phospholipid metabolism by Stp1 independently of Sre1, as this enzyme is required for phosphatidylcholine synthesis, a lipid essential for membrane structure and signaling [47]. Other downregulated genes, such as *alpha-L-arabinofuranosidase C* (linked to carbohydrate degradation) and *Pxp1* (a peroxisomal 2-hydroxyacyl-CoA lyase), suggest additional connections to cell wall remodeling and fatty acid metabolism. The most common domains in strongly regulated hypothetical protein candidates, were *centromere kinetochore component*, *major facilitator superfamily*, *fungal Zn(2)-Cys(6) binuclear cluster domain*, and *hydantoinase/oxoprolinase*, further supporting possible roles for Stp1 in membrane transport, transcriptional regulation, genome plasticity, and metabolic flexibility.

GO term enrichment analyses were consistent with the trends observed by KEGG and BLASTp analyses and revealed additional layers of functional coherence among the candidate genes. Enriched BP terms such as *glycolytic process* and *ATP synthesis coupled proton transport* among downregulated genes suggests that the absence of Stp1 may compromise central metabolic and energy-generating pathways. MF enrichment in structural constituents of *ribosomes*, *proton-transporting ATP synthase activity*, and *unfolded-protein binding*, also among downregulated genes, highlights potential impacts on protein synthesis and proteostasis. The enrichment of unfolded-protein-binding terms is particularly noteworthy because, in eukaryotes, the Stp1 homolog S2P regulates the unfolded protein response under ER stress via ATF6 [9]. This suggests that Stp1 could contribute to proper protein folding, and its downregulation may reduce the cellular capacity to manage misfolded proteins, potentially impacting ribosome biogenesis. This is supported by the observation that the *cyp61*^−^Δ*sre1*Δ*stp1* strain displayed greater sensitivity to DTT than the *cyp61*^−^ and *cyp61*^−^Δ*sre1* strains, consistent with DTT inducing ER stress, which arises from disruption of the redox environment required for disulfide bond formation during protein folding [26]. In the CC category, enrichment of *Ribosome*, further highlights a potential role of Stp1 in ribosome synthesis and protein production. Overall, these observations suggest that the downregulated genes primarily impact essential cellular processes, particularly protein synthesis. This may be related to the finding that several of the top-downregulated genes identified exclusively in *cyp61*^−^Δ*sre1*Δ*stp1* encode ribosomal proteins.

Taken together, our findings suggest that Stp1 may exert regulatory functions partially independent of Sre1 in *P. rhodozyma*, affecting multiple aspects of cellular homeostasis, including metabolism, protein folding, the response to ER stress, and ribosome biogenesis. While these connections remain to be experimentally validated, it is important to emphasize that these observations are based solely on transcriptomic data and are therefore exploratory. In this context, it is also important to note that *cyp61*^−^ and Sre1N shared many DEGs but also displayed differences. The DEGs unique to *cyp61*^−^ likely reflect physiological features intrinsic to this mutant, including its altered sterol composition and downstream metabolic consequences. Consequently, some of the transcriptional changes detected in our analyses may arise from the biology of the *cyp61*^−^ background itself rather than from the loss of Sre1 or Stp1. Therefore, the Stp1-dependent candidates identified here should be considered as context-dependent within the *cyp61*^−^ system, and additional work is needed to determine which pathways represent general features of Stp1 regulation across different growth or genetic contexts. Nevertheless, our results provide a novel framework and a strong starting point to explore previously unrecognized regulatory functions of Stp1. Clarifying these functions will require understanding the mechanistic basis of Stp1 activity, particularly because this protease must physically encounter its substrate transcription factors to initiate their activation. The environmental or metabolic conditions that could trigger such encounters remain unknown, and the growth conditions tested here may not be optimal to promote the activation mechanism of additional Stp1-dependent transcription factors. Future studies aimed at identifying such physiological signals that promote Stp1 and substrate encounters, as well as targeted validation of key candidate genes using complementary approaches, such as RT-qPCR, will be essential to strengthen and refine the exploratory findings reported here. Identifying those physiological signals will be essential to determine whether Stp1 acts on multiple, and possibly yet unidentified, membrane-bound transcription factors beyond Sre1 in *P. rhodozyma*, and to fully characterize the breadth of its regulatory influence.

## 4. Materials and Methods

### 4.1. Microorganisms and Culture Conditions

All *P. rhodozyma* strains used in this work are listed in Table 2 and their genotype was confirmed by PCR analyses before being used for RNA-seq assays (Figure S2). Strains were grown at 22 °C with constant agitation in YM medium (0.3% yeast extract, 0.3% malt extract, and 0.5% peptone) supplemented with 1% glucose.

The DH5α strain from *Escherichia coli* [48] was used for plasmid replication and maintenance, which was grown with constant agitation at 37 °C in lysogeny broth (LB) medium.

For dithiothreitol (DTT) stress assays, cell lawns were prepared on YM-1.5% agar plates, and a sterile filter disk was placed at the center. A 10 µL drop of 1.0 M DTT was applied onto the disk, and plates were incubated at 22 °C for two days.

### 4.2. P. rhodozyma Transformation

To replace the *STP1* gene with an antibiotic resistance cassette by homologous recombination in *P. rhodozyma*, plasmid pBS-Δg*STP1^nat^* [13] was used. Transformations were performed by electroporation [49,50] using a Gene Pulser Xcell^TM^ (BioRad Laboratories Inc., Hercules, CA, USA) under the following conditions: 125 mF, 600 Ω, and 0.45 kV, with 10–15 μg of linear DNA. The transformant DNA was obtained by digestion of pBS-Δg*STP1^nat^* with *Apa*I and *Xba*I to release the recombination module of 2775 bp that consists of the upstream (715 bp) and downstream (620 bp) regions of the *STP1* locus (to allow homologous recombination) and the nourseothricin resistance cassette between them inserted in opposite direction. Transformant selection was performed on 1.5% agar YM plates supplemented with 45 μg/mL nourseothricin.

### 4.3. Nucleic Acid Purification and PCR Analysis

DNA from *P. rhodozyma* was extracted by mechanically disrupting cell pellets as previously described [51]. Briefly, cell pellets were suspended in 600 μL of TE buffer (25 mM Tris-HCl, 10 mM EDTA, pH 8.0) with 100 μL of 0.5 mm glass beads and mixed with 600 μL of phenol: chloroform:isoamyl alcohol (25:24:1, *v*/*v*/*v*). The mixture was agitated using a Mini-beadbeater-16 (BioSpec Products Inc., Bartlesville, OK, USA) for three min and centrifuged for five min at 18,440× *g*. The aqueous phase was recovered and mixed with one volume of chloroform:isoamyl alcohol (24:1, *v*/*v*), followed by centrifugation for five min at 18,440× *g*. The aqueous phase was collected, and DNA was precipitated at −20 °C for one hour with one mL of cold absolute ethanol. The sample was centrifuged for 10 min at 18,440× *g*, the supernatant discarded, and the resulting DNA pellet was air-dried at 37 °C before resuspension in 100 μL of sterile water.

Plasmid DNA from *E. coli* was purified using the GeneJET Plasmid Miniprep Kit (Thermo Fisher Scientific Inc., Waltham, MA, USA) according to the manufacturer’s instructions.

PCR reactions included 1× PCR buffer (500 mM KCl, 200 mM Tris-HCl pH 8.4), 2 mM MgCl_2_, 0.2 μM each dNTP, 1 μM each primer, 1U of *Pfu* DNA polymerase, and approximately 10 ng of template DNA. Amplifications were performed in a 2720 thermal cycler (Applied Biosystems, Foster City, CA, USA) under the following conditions: initial denaturation at 94 °C for three min; 35 cycles of denaturation at 94 °C for 30 s, annealing at 55 °C for 30 s and extension at 72 °C for two min; followed by a final extension at 72 °C for 10 min and storage at 4 °C. Primers used in this work are listed in Table S1.

### 4.4. RNA Extraction, Library Preparation, and Sequencing

Three independent cultures of each strain were grown until the late exponential phase of growth (30 h of culture). The total RNA was extracted from each biological replicate, analyzed, and used for RNA-seq analyses as described below.

Cell pellets were suspended in lysis buffer (0.002 M sodium acetate, pH 5.5, 0.5% SDS, 1 mM EDTA, in 0.1% DEPC water) containing 0.5 mm glass beads. Cell disruption was performed in a Mini-beadbeater-16 for 3 min, then mixed with 800 μL of TRI Reagent^TM^ solution (Thermo Fisher Scientific Inc., Waltham, MA, USA) and followed by another 3 min of mechanical rupture. Then, chloroform was added and left at room temperature for 10 min before centrifugation at 18,440× *g* for 10 min at 4 °C. The aqueous phase was carefully collected, and RNA was precipitated using isopropanol. RNA concentration and purity were determined spectrophotometrically.

Library preparation and sequencing were performed by TCL Group (TCL Group, Santiago, Chile). Libraries were prepared using the mRNA enrichment kit VAHTS mRNA Capture Beads 2.0 (Vazyme, Nanjing, China), the directional (strand-specific) library preparation kit VAHTS Universal V8 RNA-seq Library Prep Kit for MGI (Vazyme, Nanjing, China), and the MGIEasy Circularization Kit V2.0 (MGI, Shenzhen, China). Sequencing was carried out with a DNBSEQ-G400 sequencer, 2 × 150 bp, with the MGI sequencing kit DNBSEQ-G400RS High-throughput Sequencing Set (MGI, Shenzhen, Guangdong, China), using the following adapters: Adapter Forward: AAGTCGGAGGCCAAGCGGTCTTAGGAAGACAA, Adapter Reverse: AAGTCGGATCGTAGCCATGTCGTTCTGTGAGCCAAGGAGTTG [52]. Across samples, total raw read counts ranged from 46 to 64 million (Table S3). Raw paired-end RNA-Seq reads were uploaded to the NCBI SRA database, with Accession number PRJNA966154.

### 4.5. Bioinformatic Processing and Differential Expression Analysis

Raw paired-end RNA-Seq reads were quality-filtered and adapter-trimmed using fastp v0.23.2 [53]. For each library, the average read length was estimated, and the minimum read length threshold was set adaptively according to sequencing depth (50 bp for 150 bp reads, 40 bp for 100 bp reads, and 30 bp for 75 bp reads). Low-quality bases with a Phred score < 20 were trimmed and reads failing to meet this quality requirement were discarded. Poly-G and poly-X tails were also removed to eliminate sequencing artifacts. Paired reads were synchronized to ensure mate consistency, and adapter sequences were removed. Quality control reports are summarized in Table S3.

The sequencing reads were mapped to the *P. rhodozyma* strain CBS 6938 genome (GenBank accession: GCA_014706385.1) using previously annotated gene information [19] and newly predicted genes using Augustus [54,55], implemented as a plugin in Geneious Prime 2024.0 software [56]. Gene prediction was performed using the training sets Generic and those corresponding to yeasts: *Saccharomyces*, *Cryptococcus*, *Candida albicans*, *Kluyveromyces lactis*, and *Schizosaccharomyces pombe*. The predicted coding sequences (CDS) were translated and compared against the NCBI protein database using BLASTp (Table S4). Gene matches showing ≥ 30% sequence similarity and E-values ≤ 1 × 10^−10^ were retained for functional annotation. The gff3 file with the annotation of the newly predicted genes is included as Supplementary Material (Table S5).

RNA-Seq reads were aligned to the CBS 6938 wild-type strain reference genome using Rsubread v2.18.3 [57]. Alignments were performed using the default Rsubread parameters, which include seed-and-vote mapping, Phred + 33 quality encoding, and reporting a single primary alignment per read pair. BAM files were subsequently sorted, indexed, and filtered for high-quality, properly paired reads using functions from the Rsamtools package [58]. Gene-level counts were generated using featureCounts with GFF annotation, and expression values were normalized to RPKM and TPM for each gene in each sample, producing matrices of raw counts and normalized expression for downstream analyses (Table S1). Differential gene expression analysis was performed using DESeq2 v1.40.1 [59] in R. Raw gene-level counts were imported from the count matrix generated by featureCounts. Sample conditions were assigned based on the first character of each sample name, and all pairwise comparisons between conditions were conducted. For each comparison, a DESeqDataSet was constructed, with the design formula ~ Condition, and the reference condition was specified to ensure consistent contrast direction. The DESeq2 workflow was executed to estimate size factors, dispersion, and negative binomial model parameters, followed by Wald tests to identify differentially expressed genes. Differential expression was defined using a log_2_ fold-change threshold of ±1 and an adjusted *p*-value (padj) ≤ 0.05. Genes meeting both criteria were classified as upregulated (log_2_FC ≥ 1, padj ≤ 0.05) or downregulated (log_2_FC ≤ −1, padj ≤ 0.05). Results for each condition pair are shown in Table S6.

Gene classification into metabolic pathways and functional modules was performed using the KEGG Automatic Annotation Server, KAAS, [60], with the default GHOSTX parameters [61] and eukaryotic gene datasets. GO enrichment analysis was performed using both topGO [62] with the elim algorithm and Fisher’s exact test, reporting elimFisher *p*-values, which accounts for the GO hierarchy to highlight specific terms, and clusterProfiler [63] with a hypergeometric test and Benjamini–Hochberg correction, providing a broader overview of enriched functional categories for comparison. Using both approaches helps minimize method-specific biases, yielding more reliable and biologically coherent enrichment results. Custom GO terms annotations were obtained using the Blast2GO suite, which incorporates InterProScan to transfer GO terms based on protein domain information [64].

Plots were generated in Python 3 using pandas (version 2.2.3) [65], Matplotlib (version 3.9.2) [66], seaborn (version 0.13.2) [67], and NumPy (version 2.1.3) [68] packages.

## 5. Conclusions

Our transcriptomic analyses in both wild-type and *cyp61*^−^ genetic backgrounds revealed that Stp1’s regulatory role primarily functions through the Sre1 transcription factor, as expected. However, several genes were differentially expressed in the Δ*stp1* mutants that were independent from Sre1, indicating additional regulatory roles for Stp1. These genes were linked to amino acid metabolism, protein synthesis, redox balance, central carbon metabolism, and stress adaptation, suggesting that Stp1 may exert a broader regulatory role in *P. rhodozyma* beyond regulation of sterol homeostasis. Nevertheless, the conditions tested in this study may not capture the environmental or metabolic signals required for initiating the activation of potential Stp1-dependent transcription factors to promote their encounter. Defining these conditions will be crucial to uncover the full extent of Stp1-mediated regulation and to determine whether Stp1 functions as a protease that activates multiple and probably yet unidentified transcription factors besides Sre1 in *P. rhodozyma*.

## Figures and Tables

**Table 1:** GO terms significantly enriched in common between topGO and clusterProfiler.

				clusterProfiler		topGO		
GO Term	Description	DEGs	Ontology	Count	*p*.Adjust	Annotated	Significant	*p*-Value (elimFisher)
GO:0006096	Glycolytic process	Dw	BP	2	0.0319	13	2	0.008
GO:0015986	ATP synthesis coupled proton transport	Dw	BP	2	0.0385	15	2	0.011
GO:0005840	Ribosome	Dw	CC	5	0.0104	131	6	0.00046
GO:0005730	Nucleolus	Up	CC	5	0.0084	78	5	0.004
GO:0003735	Structural constituent of ribosome	Dw	MF	6	0.0464	125	6	0.0025
GO:0046933	Proton-transporting ATP synthase activity	Dw	MF	2	0.0464	10	2	0.0053
GO:0051082	Unfolded protein binding	Dw	MF	3	0.0464	34	3	0.0063

GO term: identifier of the Gene Ontology term. Description: descriptive name of the GO term. Ontology: corresponding ontology category—Biological Process (BP), Molecular Function (MF), or Cellular Component (CC). DEGs: differentially expressed genes, Up: Upregulated, Dw: Downregulated. Count (clusterProfiler): number of DEGs assigned to each GO term according to clusterProfiler. *p*.adjust (clusterProfiler): multiple-testing adjusted *p*-value (Benjamini–Hochberg) according to clusterProfiler. Annotated (topGO): total number of genes in the universe annotated with each GO term in topGO. Significant (topGO): number of DEGs assigned to each GO term in topGO. *p*-value (elimFisher, topGO): hierarchical *p*-value obtained using the elimFisher method in topGO.

**Table 2:** Yeast strains used in this work.

Strains	Description	Reference
CBS 6938	Wild-type strain, from which all mutants derive.	ATCC 96594
CBS.*FLAG*.*SRE1*	Strain WTF. Mutant Zeo^S^, Hyg^R^ and Ntc^S^ that derives from the *P. rhodozyma* wild-type strain CBS 6938 (ATCC 96594). The gene *SRE1* was replaced by a gene variant that expresses the Sre1 protein fused to the 3xFLAG epitope at its N-terminus, followed by the hygromycin B resistance cassette.	[13]
CBS.*sre1*^-^	Strain Δ*sre1.* Mutant Zeo^R^, Hyg^S^ and Ntc^S^. Approximately 90% of the coding region of gene *SRE1* was replaced by the zeocin resistance cassette.	[12]
CBS.*FLAG.SRE1.*∆*stp1*	Strain Δ*stp1.* Mutant Zeo^R^, Hyg^R^ and Ntc^S^. The *STP1* locus was replaced by the hygromycin B resistance cassette. The gene *SRE1* was replaced by a gene variant that expresses the Sre1 protein fused to the 3xFLAG epitope at its N-terminus, followed by the zeocin resistance cassette.	[13]
CBS.*sre1*^-^.∆*stp1*	Strain Δ*sre1*Δ*stp1.* Mutant Zeo^R^, Hyg^S^ and Ntc^R^. Approximately 90% of the coding region of gene *SRE1* was replaced by the zeocin resistance cassette. The *STP1* locus was replaced by the nourseothricin resistance cassette.	This work
CBS.*FLAG.SRE1N*	Strain Sre1N. Mutant (Zeo^R^, Hyg^S^ and Ntc^S^). The *SRE1* gene was replaced by a gene version that expresses Sre1N fused to the 3xFLAG epitope at its N-terminal, followed by the zeocin resistance cassette.	[12]
CBS*.cyp61^−^.FLAG.SRE1*	Strain *cyp61*^−^. Mutant Zeo^R^, Hyg^R^ and Ntc^S^. The *CYP61* locus was interrupted by the zeocin resistance cassette. The native *SRE1* gene was replaced by a gene variant that expresses the Sre1 protein fused to the 3xFLAG epitope at its N-terminus, followed by the hygromycin B resistance cassette.	[13]
CBS.*cyp61^−^*.*sre1*^-^	Strain *cyp61*^−^Δ*sre1.* Mutant Zeo^R^, Hyg^R^ and Ntc^S^. The *CYP61* locus was interrupted by the hygromycin B resistance cassette. Approximately 90% of the coding region of gene *SRE1* was replaced by the zeocin resistance cassette).	[12]
CBS.*cyp61^−^.FLAG.SRE1.*∆*stp1*	Strain *cyp61^−^*Δ*stp1*. Mutant Zeo^R^, Hyg^R^ and Ntc^R^. The *CYP61* locus was interrupted by the zeocin resistance cassette. The *STP1* locus was replaced by the nourseothricin resistance cassette. The *SRE1* gene was replaced by a variant that expresses the Sre1 protein fused to the 3xFLAG epitope at its N-terminus, followed by the hygromycin B resistance cassette.	[13]
CBS.*cyp61^−^*.*sre1^−^.*∆*stp1*	Strain *cyp61^−^*Δ*sre1*Δ*stp1.* Mutant Zeo^R^, Hyg^R^ and Ntc^R^. The *CYP61* locus was interrupted by the hygromycin B resistance cassette. Approximately 90% of the coding region of gene *SRE1* was replaced by the zeocin resistance cassette). The *STP1* locus was replaced by the nourseothricin resistance cassette.	This work

Zeo^S^/Zeo^R^: sensitive/resistant to zeocin. Hyg^S^/Hyg^R^: sensitive/resistant to hygromycin B. Ntc^S^/Ntc^R^: sensitive/resistant to nourseothricin. ATCC American Type Culture Collection.

## Data Availability

The datasets generated and analyzed during this study are available at the National Center for Biotechnology Information SRA database (Accession number PRJNA966154) and as Supplementary Materials.

## Supplementary Materials

The following supporting information can be downloaded at: https://www.mdpi.com/article/10.3390/ijms262412008/s1.

## Abbreviations

The following abbreviations are used in this manuscript:

CDS	Coding sequences
DEGs	Differentially Expressed Genes
ER	Endoplasmic Reticulum
GO	Gene Ontology
KEGG	Kyoto Encyclopedia of Genes and Genomes
S1P	Site-1 Protease
S2P	Site-2 Protease
SCAP	SREBP Cleavage-Activating Protein
SRE	Sterol Regulatory Element
SREBP	Sterol Regulatory Element-Binding Protein
