# Supplementary figures and images for: Comparative Transcriptomic Analyses Reveal Potential Stp1 Regulatory Roles Independent of Sre1 in Phaffia rhodozyma
Source: Int J Mol Sci. 2025 Dec 13;26(24):12008. doi: 10.3390/ijms262412008 (PMC12732919; doi:10.3390/ijms262412008)

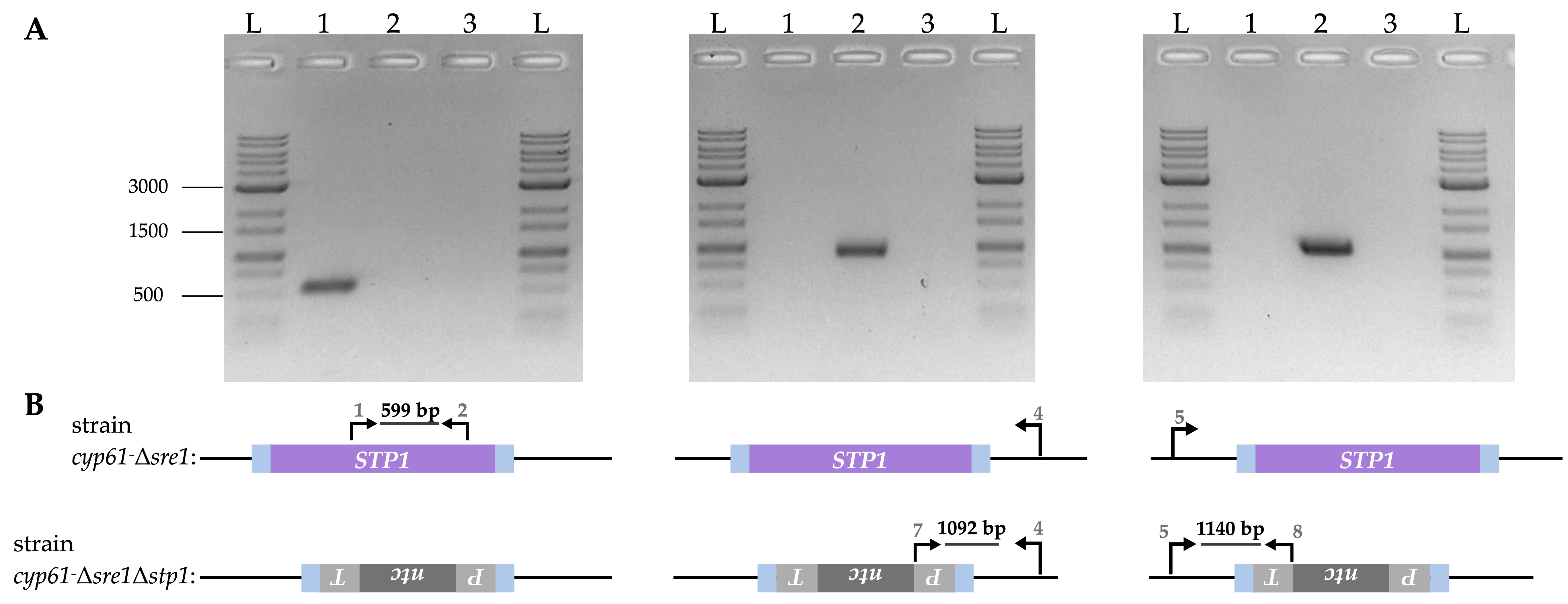

Supplement: Supplementary file 1 [file ijms-26-12008-s001.zip › Figure S1.jpg]

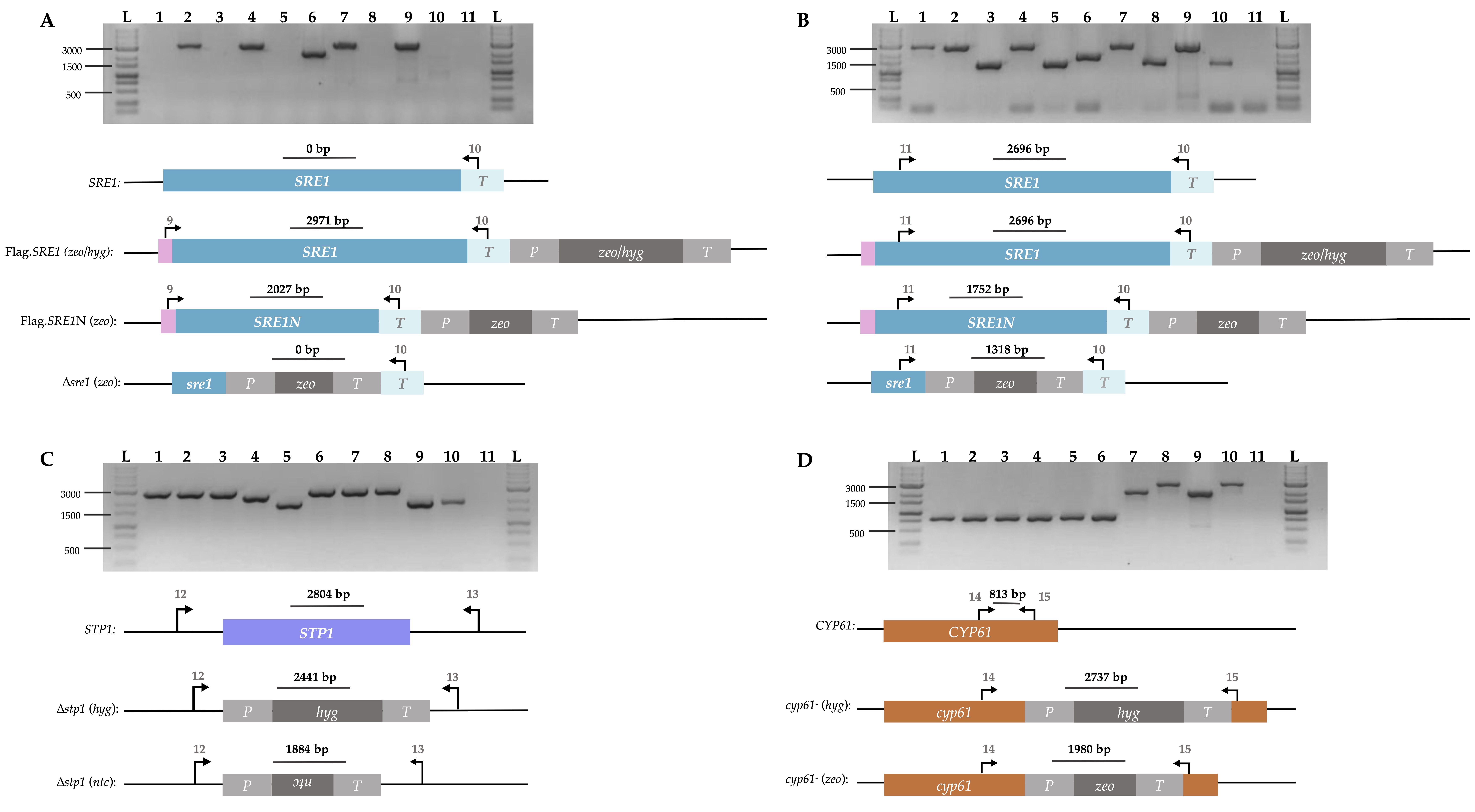

Supplement: Supplementary file 1 [file ijms-26-12008-s001.zip › Figure S2.jpg]

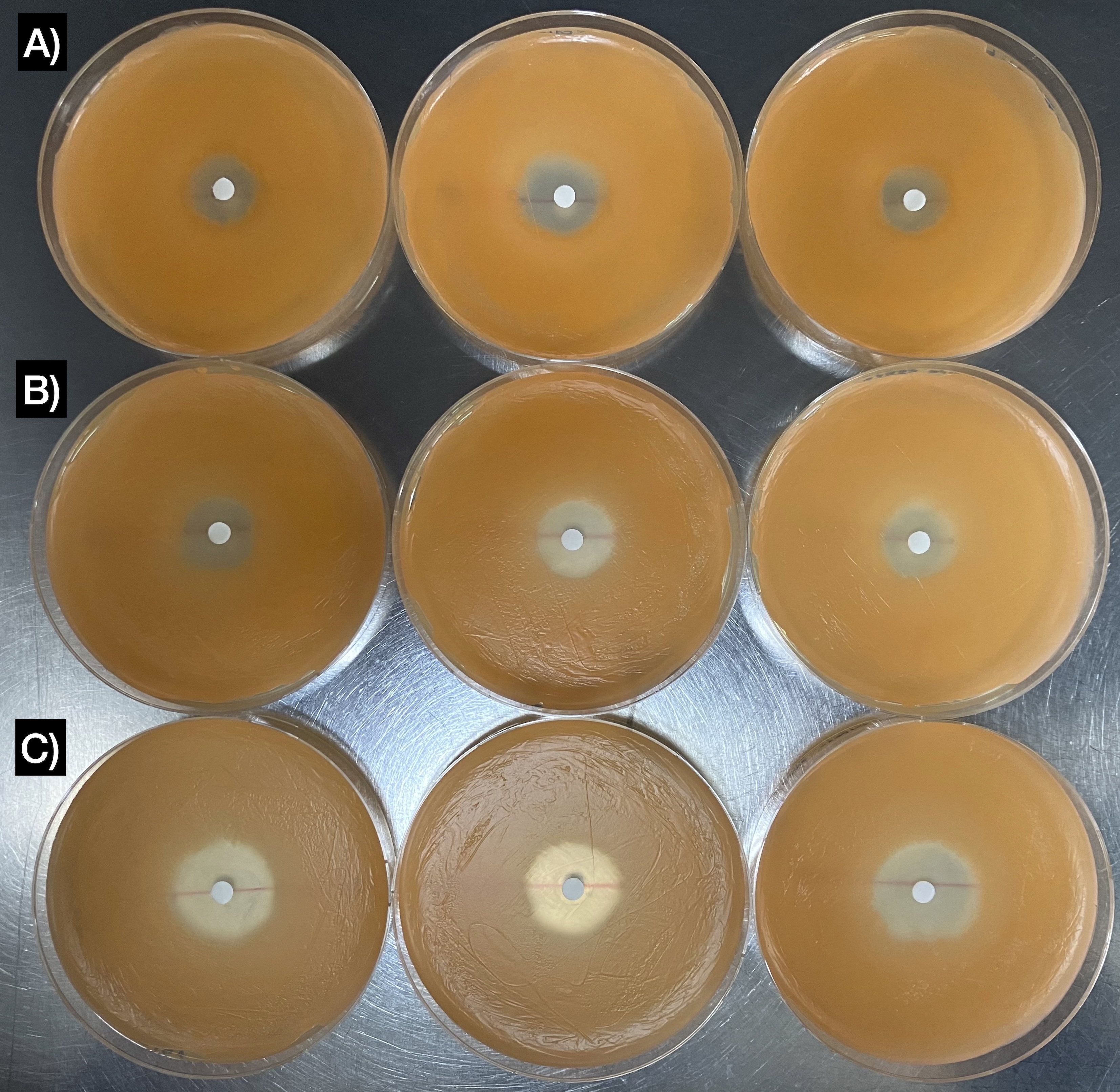

Supplement: Supplementary file 1 [file ijms-26-12008-s001.zip › Figure S3.jpg]
